# Unexplored Roles of Erythrocytes in Atherothrombotic Stroke

**DOI:** 10.3390/neurolint15010011

**Published:** 2023-01-23

**Authors:** Charalampos Papadopoulos, Konstantinos Anagnostopoulos, Dimitrios Tsiptsios, Stella Karatzetzou, Eirini Liaptsi, Irene Zacharo Lazaridou, Christos Kokkotis, Evangelia Makri, Maria Ioannidou, Nikolaos Aggelousis, Konstantinos Vadikolias

**Affiliations:** 1 Laboratory of Biochemistry, Department of Medicine, Democritus University of Thrace, 68100 Alexandroupolis, Greece; 2 Department of Neurology, Democritus University of Thrace, 68100 Alexandroupolis, Greece; 3 Department of Physical Education and Sport Science, Democritus University of Thrace, 69100 Komotini, Greece

**Keywords:** erythrocyte, ischemic stroke, atherothrombosis, lipotoxicity, glucotoxicity, homocysteine, immunometabolism, neuroinflammation

## Abstract

Stroke constitutes the second highest cause of morbidity and mortality worldwide while also impacting the world economy, triggering substantial financial burden in national health systems. High levels of blood glucose, homocysteine, and cholesterol are causative factors for atherothrombosis. These molecules induce erythrocyte dysfunction, which can culminate in atherosclerosis, thrombosis, thrombus stabilization, and post-stroke hypoxia. Glucose, toxic lipids, and homocysteine result in erythrocyte oxidative stress. This leads to phosphatidylserine exposure, promoting phagocytosis. Phagocytosis by endothelial cells, intraplaque macrophages, and vascular smooth muscle cells contribute to the expansion of the atherosclerotic plaque. In addition, oxidative stress-induced erythrocytes and endothelial cell arginase upregulation limit the pool for nitric oxide synthesis, leading to endothelial activation. Increased arginase activity may also lead to the formation of polyamines, which limit the deformability of red blood cells, hence facilitating erythrophagocytosis. Erythrocytes can also participate in the activation of platelets through the release of ADP and ATP and the activation of death receptors and pro-thrombin. Damaged erythrocytes can also associate with neutrophil extracellular traps and subsequently activate T lymphocytes. In addition, reduced levels of CD47 protein in the surface of red blood cells can also lead to erythrophagocytosis and a reduced association with fibrinogen. In the ischemic tissue, impaired erythrocyte 2,3 biphosphoglycerate, because of obesity or aging, can also favor hypoxic brain inflammation, while the release of damage molecules can lead to further erythrocyte dysfunction and death.

## 1. Introduction

Stroke constitutes the second highest cause of morbidity and mortality worldwide while also impacting the world economy, triggering substantial financial burden on national health systems. The incidence of stroke, and ischemic stroke in particular, constantly progresses. In 2010, 11.6 million people suffered a new ischemic stroke, while in 2016, this number increased to 13.7 million. Meanwhile, the prevalence of stroke further underlines the necessity for basic research: in 2016, 80.1 million people suffered a stroke at least once. Regarding mortality, 5.5 million deaths were attributed to stroke in 2016. Another determinant of the impact of the disease on the human population, disability-adjusted life-years (DALY), is also alarming: 116 million DALYs were lost in 2016. Although the fatality of stroke seems to be declining, its mortality is increasing [1]. These facts strongly suggest that despite the existing interventions for stroke, such as intravenous thrombolysis and mechanical thrombectomy, strokes continue to negatively influence human lives and economies. Hence, there is an urgent need for the discovery of novel therapeutic targets.

Atherothromboses comprise 37% of all ischemic strokes [2]. Atherothrombotic strokes are formed when a blood clot blocks a brain artery. This clot is previously formed by a ruptured atherosclerotic plaque. Rupture takes place when a plaque is unstable due to endothelial dysfunction, inflammation, and a large necrotic lipid core [3].

**Figure 1:**
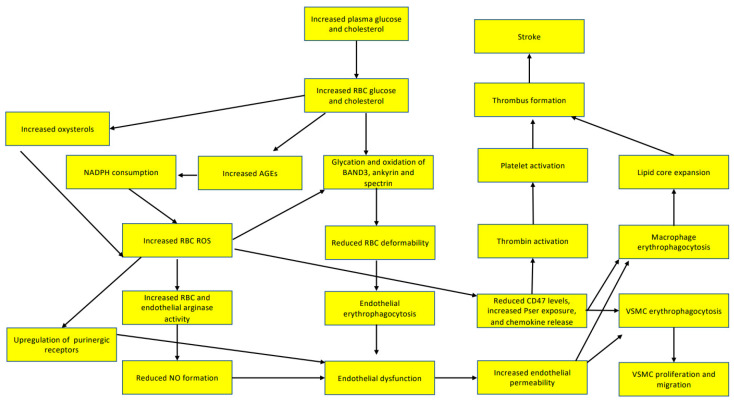
Risk factors for atherothrombosis, such as dyslipidemia and hyperglycemia, act on circulating red blood cells. Both high glucose and high cholesterol result in impaired erythrocyte antioxidant capacity. This leads to protein and lipid oxidation, subsequently lowering membrane deformability. In addition, reactive oxygen and nitrogen species drive arginase activity upregulation and programmed erythrocyte death (eryptosis). Erythrocytes are recognized by endothelial cells through their exposed phosphatidylserine and advanced glycation end products. In addition, erythrocyte-derived reactive oxygen species (ROS) upregulate purinergic receptors on endothelial cells. These events lead to endothelial activation and permeability. Permeable and activated endothelial cells allow monocytes and neutrophils to enter the arterial wall. Red blood cells also infiltrate and are phagocytized by macrophages and vascular smooth muscle cells. The release of chemokines and sphingosine 1-phosphate, along with exposed phosphatidylserine and reduced cluster of differentiation 47 (CD47) membrane levels, drive erythrophagocytosis. High cholesterol levels in erythrocytes facilitate the formation of foam cells, contributing to the expansion of the necrotic lipid core, while vascular smooth muscle cells (VSMCs) migrate and multiply. Exposed phosphatidylserine contributes to platelet activation, while erythrocyte CD47 molecules interact with fibrinogen. These events lead to atherothrombosis. Atherothrombosis restrains oxygen supply to the brain, causing an ischemic stroke. CD47: cluster of differentiation 47; NO: nitric oxide; RBC: red blood cells; ROS: reactive oxygen species; VSMCs: vascular smooth muscle cells The Figure was partly generated using Servier Medical Art, provided by Servier and licensed under a Creative Commons Attribution 3.0 unported license.

High levels of blood glucose [4], homocysteine [5], and cholesterol [6] are causative factors for atherothrombosis. These molecules highly impact erythrocyte function. Here, we describe the molecular mechanisms by which erythrocytes contribute to the different steps of atherothrombosis, possibly by mediating the effects of glucose, homocysteine, and cholesterol, which are shown in Figure 1.

Despite the deciphering of the above-mentioned molecular mechanisms, there is still a need for further untangling of the molecular mechanisms of stroke progression.

## 2. Red Blood Cells Participate in Innate Immunity

In the last decades, a merge of immunology and biochemistry–molecular biology has emerged, which has been termed “immunometabolism”. Through a detailed examination of the metabolic regulation of immune cells and the regulation of metabolism by immune-derived molecules, researchers have managed to explain the molecular basis of various diseases with immune and metabolic components. Diabetes mellitus, infections, non-alcoholic fatty liver disease, atherosclerosis, etc., are now known to be controlled by a disrupted cross-talk between immunity and metabolism [7]. Innate immunity is comprised, apart from classical immune cells, of endothelial cells and erythrocytes. Red blood cells scavenge chemokines, reactive oxygen species (ROS), mitochondrial DNA, and complement proteins from the circulation and release ROS, hemoglobin, chemokines, and cytokines, as well as microvesicles, while also interacting with immune cells through surface molecules. In addition, erythrocytes synthesize and release bioactive lipids and participate in the reverse cholesterol transport [8]. Furthermore, erythrocytes constitute a link between the systemic metabolic status and immune function [9]. These activities of red blood cells are largely regulated by extracellular signals, such as lipids, hormones, glucose, and other metabolites, in the circulation or the microenvironment they are found in [10,11,12,13].

## 3. Red Blood Cells in Atherosclerosis

### 3.1. Red Blood Cells Participate in Endothelial Dysfunction

Reactive oxygen species, along with thrombin-activated endothelial cells, through the expression of P-selectin, E-selectin, intercellular adhesion molecule 1 (I-CAM), and the vascular cell adhesion protein (V-CAM), allow the adherence of neutrophils, monocyte-derived macrophages, T cytotoxic cells, T helper cells, T regulatory cells, γδ Τ cells, mast cells, platelets, natural killer cells, and dendritic cells. In addition, reduced nitric oxide production also leads to the upregulation of adhesive molecules by endothelial cells. Apart from adhesive molecules, endothelial cells contribute to plaque infiltration by leukocytes through the secretion of various chemotactic molecules [14].

Erythrocytes present signs of glucotoxicity, which markedly impacts endothelial function. High glucose levels disrupt the anti-oxidant capacity of red blood cells, an event associated with several erythrocyte functions, as discussed below. Red blood cells exposed to high concentrations of glucose in vitro or in vivo heavily impact endothelial function. Glycated erythrocytes present advanced glycation end products (AGEs). These molecules allow for the interaction between red blood cells and endothelial cells through the receptor for AGEs. This results in the nuclear factor kappa-light-chain-enhancer of activated B cell (NF-κB)-dependent endothelial inflammation [15]. In addition, glycated erythrocytes interact with endothelial cells through their exposed phosphatidylserine. The recognition of this apoptotic signal drives erythrophagocytosis, culminating in the reduced proliferation and migration of endothelial cells. In vivo, this interaction was proved to be part of the atherothrombotic environment [16]. Erythrocytes can also disrupt the normal functioning of endothelial cells through the disrupted release of nitric oxide. Increased reactive oxygen species in red blood cells through post-translational modifications result in augmented activity and expression of arginase-1 [17]. Furthermore, erythrocyte-derived ROS also upregulate endothelial arginase and purinergic receptors. The activation of these receptors by adenosine triphosphate (ATP) leads to further formation of ROS by endothelial cells [18]. Since the half-life of ROS is relatively short to mediate a cell-to-cell interaction, we speculate that the propagation of free radicals mediates the erythrocyte-derived ROS effect on endothelial cells. It is likely the oxidized phospholipids that ultimately interact with endothelial cells [19]. Apart from ROS, erythrocyte arginase is also positively regulated by peroxynitrite, which can be formed by nitric oxide synthase, when arginine is not available [20]. Arginase and nitric oxide synthase compete for arginine. Hence, increased erythrocyte arginase and erythrocyte-induced endothelial arginase depletes arginine for the synthesis of nitric oxide. The end result could be the increased expression of adhesive molecules. Reduced miR-210 levels in erythrocytes from type 2 diabetes patients also drive endothelial dysfunction [21].

Interestingly, increased glucose can lead to sorbitol production in erythrocytes, impairing the antioxidant function of red blood cells [22]. However, increased erythrocyte sorbitol levels have been associated with positive outcomes in stroke patients [23]. Although the mechanism explaining this discrepancy is not known, we can only speculate that NADPH consumption for sorbitol production may limit the pool of NADPH for NADPH oxidase, thus limiting the production of ROS. Presumably, ROS production by NADPH oxidase may act on proteins and lipids in the erythrocyte that are different from those that are affected by decreased ROS neutralization due to sorbitol production.

Apart from glucotoxicity, erythrocyte function is disrupted by lipotoxicity [10]. Mahdi et al. [24] recently reported that dyslipidemia resulted in erythrocyte oxidative stress. These erythrocytes could then upregulate the activity of arginase-1 of endothelial cells. Similar results were reported earlier by Unruh et al. [25]. They showed that a high-fat diet led to erythrocyte phosphatidylserine exposure, chemokine binding and release, cholesterol accumulation, and increased ROS. In that study, they also showed induced endothelial activation. Hypercholesterolemia also induces eryptosis and the adherence of erythrocytes to the endothelium [26]. This effect can be attributed to increased hypercholesterolemia-induced oxysterols. Oxysterols trigger oxidative stress-dependent phosphatidylserine exposure [27]. Mechanistically, 7-keto-cholesterol activates RAC-GTPase and protein kinase C ζ (PKCζ), leading to the activation of nicotinamide adenine dinucleotide phosphate (NADPH) oxidase. The 6-beta triol leads to the activation of nitric oxide synthase-dependent nitrosative stress [28].

Decreased erythrocyte deformability facilitates erythrophagocytosis by endothelial cells. It is worth noting that arginase activation results in polyamine synthesis. Polyamines have been shown to decrease erythrocyte deformability [29]. Other researchers have shown that oxidative stress-induced erythrocyte phosphatidylserine exposure and reduced deformability drive endothelial cell erythrophagocytosis [30,31]. Brain endothelial erythrophagocytosis then drives hemoglobin transmigration to the brain, resulting in endothelial cell death [32]. Although endothelial erythrophagocytosis can also have an effect on the periphery, as already discussed, in the brain, it can exert direct effects, due to the transmigration of hemoglobin.

Apart from hyperglycemia and dyslipidemia, erythrocytes are vulnerable to hyperhomocysteinemia. Homocysteine inhibits several antioxidant enzymes of erythrocytes while also inhibiting the degradation of asymmetric dimethylarginine (ADMA), thus increasing its levels. ADMA can next inhibit the synthase of nitric oxide and upregulate the activity of arginase [33,34]. Homocysteine conversion to adenosylhomocysteine inhibits methyltransferases, and this can lead to the reduction of the erythrocyte’s kynurenic acid (KA) [35]. KA is essential for the normal functioning of brain endothelial cells. Homocysteine can result in erythrocyte phosphatidylserine exposure and microvesicle release [36].

In summary, high glucose, cholesterol, and homocysteine levels in circulation result in increased erythrocyte ROS. This event leads to arginase upregulation, phosphatidylserine exposure, and reduced deformability. These mechanisms deplete nitric oxide from endothelial cells and induce endothelial erythrophagocytosis. The final result is endothelial activation.

### 3.2. Red Blood Cells Participate in Lipid Core Expansion of Atherosclerotic Plaques

The accumulation of cholesterol crystals in the atherosclerotic lesion makes the plaque vulnerable to rupture [6]. Cholesterol crystals can be derived from lipoproteins, mainly low-density lipoproteins, which are oxidized when trapped inside the arterial walls. However, intraplaque hemorrhage permits access to erythrocytes for the plaque’s lipid core. Tziakas et al., through multiple studies, showed that erythrocyte cholesterol positively affected both the burden and the clinical instability of the atherosclerotic plaque [37,38,39,40,41,42,43]. In particular, they discovered that both the free- and esterified cholesterols of erythrocyte membranes contributed to the expansion and instability of the atheromatic lipid core.

### 3.3. Red Blood Cells Participate in the Inflammatory Activation of Macrophages in Atherosclerotic Plaques

In the atherosclerotic plaque, macrophages contribute to the inflammatory state by secreting cytokines, such as interferon-γ (IFN-γ), which induces the expression of collagen. Macrophages also degrade the extracellular matrix through the secretion of metalloproteinases and phagocytize cellular debris and oxidized lipoproteins contributing to the lipid core. Through these actions, macrophages render the plaque unstable. They also contribute to thrombosis through the expression of tissue factor [3].

Wang et al. found that reduced erythrocyte cluster of differentiation 47 (CD47) levels drove macrophage erythrophagocytosis in atherosclerosis. Interestingly, they showed that macrophage erythrophagocytosis was mainly located in the necrotic core, hence favoring plaque instability. They also reported that erythrophagocytosis resulted in the inflammatory response of macrophages and defected efferocytosis. Tziakas et al. previously showed that erythrocyte interleukin 8 (IL-8) levels were clinically associated with unstable atherosclerotic plaque [44]. Similar results were later reported by Unruh et al. In their study, they found increased erythrocyte cholesterol, IL-8, monocyte chemoattractant protein 1 (MCP1), reactive oxygen species (ROS), and exposed phosphatidylserine in animals that were fed a high-fat diet. These characteristics enhanced macrophage erythrophagocytosis and subsequent adhesion to the endothelium [25].

### 3.4. Red Blood Cells Participate in Vascular Smooth Muscle Cell Function

In the early stages of atherosclerotic plaque formation, erythrocytes can reach the vascular wall and interact with smooth muscle cells [3]. This leads to erythrophagocytosis and consequently, to the intracellular accumulation of iron, heme, and lipids [45]. Later studies showed that erythrophagocytosis is driven by the externalized phosphatidylserines of erythrocytes, which is recognized by the milk fat globule-EGF factor 8 protein (MGF-E8). Upregulation of the MGF-E8 is controlled by erythrocyte-derived sphingosine 1-phosphate (S1P)-dependent S1PR2 activation [46].

### 3.5. Red Blood Cells Participate in T-Cell Activation

Erythrocyte antioxidant capacity can halter T-cell activation. However, under the oxidant environment of atherothrombosis, erythrocytes are unable to neutralize the ROS related to T-cell activation [47,48]. This incapability of controlling T-cell activation can be provoked by high glucose, homocysteine, and other metabolites.

## 4. Red Blood Cells Participate in Thrombus Formation

### 4.1. Red Blood Cells Determine Blood Viscosity

Erythrocytes possess a unique cytoskeleton, which allows for rapid alterations in both shape and size. This cytoskeleton is characterized by the interaction of the membrane protein Band3 with the proteins ankyrin and protein band 4.1, which are attached to the spectrin dimer. In vitro, the exposure of red blood cells to high glucose concentrations or erythrocytes from diabetic patients leads to reduced deformability. This impaired function of red blood cells is triggered by the oxidation of Band3 [49], as well as ankyrin, protein band 4.1, and spectrin [50]. Mechanistically, the oxidation of Band3 induces the formation of clusters in the membrane, which limit its association with the cytoskeleton and its lateral diffusion. The oxidation of the cytoskeletal proteins further amplifies this event. Consequently, reduced erythrocyte deformability leads to an increase in the blood viscosity, facilitating the formation of thrombus [51].

### 4.2. Red Blood Cells Participate in Neutrophil Extracellular Traps of Atherothrombotic Lesions

Neutrophil extracellular traps (NET) are an antimicrobial mechanism of neutrophilic granulocytes. Their main component is extracellular DNA that is associated with antimicrobial proteins [52]. NETosis is a type of cell death dependent on the formation of NETs. It actively contributes to atherothrombosis through the increased disposition of interleukin 17 (IL-17) and tissue factor. In addition, NETosis activates macrophages, resulting in increased T helper cell 17 (Th17) levels [53]. Recently, Chilingaryan et al. [54] reported that erythrocytes can be found in the NETs of atherothrombotic lesions. More importantly, they showed that these erythrocytes were largely fragmented and constituted the majority of the atherothrombotic lesions.

### 4.3. Red Blood Cells Participate in Platelet Activation

The activation of platelets in ruptured plaques is the first step for the formation of atherothrombosis [3]. Erythrocytes can lead to platelet activation through prothrombin activation. In particular, erythrocyte phosphatidylserine exposure functions as a platform for the activation of prothrombin. This pro-coagulant function of red blood cells can be triggered by the actions of lysophosphatidic acid [55], thromboxane 2, arachidonic acid [56], homocysteine [36], and the activation of the FAS cell surface death receptor (FASR) by the platelet FAS ligand (FASL) [57]. In addition, erythrocyte–platelet interaction upregulates the exposure of P-selectin and a2bβ3 integrin on platelets, which can further enhance leukocyte recruitment at the atherothrombotic site. Furthermore, erythrocyte-derived adenosine triphosphate (ATP) can also lead to platelet nitric oxide (NO) production [58], while erythrocyte-derived adenosine diphosphate (ADP) leads to platelet aggregation [59].

## 5. Red Blood Cells Participate in Thrombus Stabilization

The previous steps are necessary for rendering atherosclerotic plaques unstable and provoking platelet activation and clot formation. However, not every atherothrombosis results in artery occlusion and hence cerebral stroke. For artery occlusion to take place, it requires thrombus propagation. Thrombi containing red blood cells are larger [60]. The incorporation of erythrocytes in thrombi is dependent on erythrocyte–fibrinogen interactions. These interactions are formed by binding fibrinogen to the β3 integrin [61] and CD47 receptor of the erythrocyte [62].

However, erythrocyte-rich thrombi have been associated with favorable outcomes in patients being treated with mechanical thrombectomy [63]. Since erythrocyte vesiculation shreds CD47 from the erythrocyte surface [64], it would be interesting to examine the role of lipid metabolism on erythrocyte–fibrinogen interactions.

## 6. Red Blood Cells Participate in Hypoxia after Atherothrombotic Stroke

Erythrocytes regulate oxygen release. This function is mainly regulated by the formation of the glycolytic intermediate 2,3 biphosphoglycerate (2,3-BPG). This metabolite is formed by biphosphoglycerate mutase and results in the lower oxygen affinity of hemoglobin. The activity of mutase in erythrocytes increases after exposure to hypoxia. Its levels of activity are regulated by transglutaminase, which stabilizes mutase [65]. In addition, the formation of 2,3-BPG is favored when glycolysis is upregulated. In red blood cells under hypoxia, this is mediated by adenosine receptor 2b-mediated sphingosine kinase 1 activity, and the subsequent sphingosine-1-phosphate (S1P)-induced glycolytic enzyme is release from the plasma membrane to the cytoplasm [66]. In addition, erythrocyte adenosine receptor 2b activation during hypoxia also results in adenosine monophosphate-dependent kinase (AMPK) activation and AMPK-dependent BPG mutase [67]. Furthermore, S1P increase also enhances AMPK activation and subsequent BPG mutase elevated activity by downregulating the activity of the pp2a phosphatase, which negatively controls AMPK [68]. Increased levels of erythrocyte 2,3-BPG have been shown to protect from ischemia [69], while the beneficial effects of remote ischemic conditioning are attributed to increased erythrocyte 2,3-BPG [69].

**Figure 2:**
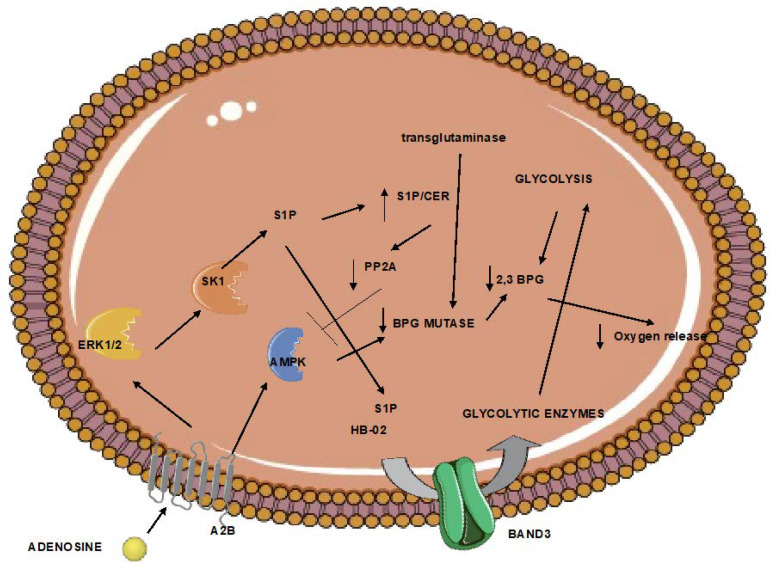
Erythrocytes trapped in the ischemic tissue can respond to limited oxygen through the regulation of the 2,3-bisphosphoglycerate. However, aging and obesity halter this pathway. The end result can be increased brain inflammation. (A normal arrow signifies activation. An arrow with a vertical line signifies inhibition). 2,3 BPG: 2,3 biphosphoglycerate; A2B: adenosine receptor 2B; AMPK; adenosine monophosphate-activated kinase, BPG mutase: biphosphoglycerate mutase; CER: ceramide; ERK1/2: extracellular signal-regulated kinase; HB-O2: oxygenated hemoglobin; S1P: sphingosine-1 phosphate; SK1: sphingosine kinase 1.

However, obesity and aging have been associated with reduced 2,3-BPG levels [70] and BPG mutase activity [66], respectively (Figure 2). Animals lacking erythrocyte adenosine receptor a2b, and consequently, BPG mutase activity, present markers of brain inflammation.

## 7. Red blood Cells Respond to Damage-Associated Molecular Patterns Released after Ischemic Stroke

Ischemia and sustained oxygen deprivation of the brain can lead to the release of damage-associated molecular patterns (DAMPs), such as ATP, extracellular histones, amyloids, and cell-free mitochondrial DNA. DAMPs can act on microglial cells and infiltrate immune cells. This event regulates both brain inflammation and tissue repair [71]. Apart from DAMPs, pathogen-associated molecular patterns (PAMPs) are also implicated in the progression of stroke; lipopolysaccharide (LPS), a component of Gram-negative bacteria, is associated with poor prognosis in stroke [72]. Apart from immune cells, DAMPs can also act on red blood cells (Table 1). Erythrocytes express various protein receptors that are capable not only of scavenging but also sensing DAMPs and PAMPs. In addition, as analyzed below, several DAMPs and PAMPs trigger physicochemical alterations on red blood cells. The net effect of DAMP-induced erythrocyte dysfunction is mainly determined by the effect of erythrophagocytosis on macrophage polarization. While in the liver erythrolysis prior to erythrophagocytosis results in an anti-inflammatory phenotype of macrophages [73], in the brain, this mechanism induces inflammation [74]. We speculate that the relative quantities of CD47, oxidized CD47, exposed phosphatidylserine, and the level of ROS determine the ratio of erythrolysis: intact erythrophagocytosis.

### 7.1. Cell-Free Mitochondrial DNA and CpG DNA

Hotz et al. [75] were the first to report that human erythrocytes expressed the toll-like receptor 9 (TLR9) on their plasma membranes. In their study, they showed that TLR9 on erythrocyte membranes bound cell-free mitochondrial DNA (mtDNA). In fact, they reported that when the circulating levels of mtDNA were low, most TLR9s of erythrocytes had bound mtDNA. However, an increase in the circulating levels of mtDNA resulted in the saturation of the scavenging capacity of red blood cells. In addition, the loss of TLR9 on red blood cells led to increased lung injury, while the administration of red blood cells from healthy donors attenuated the CpG DNA-induced inflammation.

Subsequent reports from the same research group provided important clues for the effects of cell-free mitochondrial DNA and CpG DNA, in general, on erythrocytes. First, red blood cells from septic patients contained increased TLR9 and bound CpG DNA levels in comparison to healthy controls [76]. Furthermore, CpG DNA was found to induce morphological changes on erythrocytes in a TLR9-dependent manner. Specifically, a redistribution of BAND3 was observed, as well as a conformational change and the loss of the “do not eat me” signaling protein CD47. It was also remarkable that CpG DNA binding to TLR9 brought about a marked increase in the levels of TLR9 on the surface of erythrocytes. Finally, it was shown that CpG DNA binding to TLR9 augmented erythrophagocytosis and inflammation in vivo.

These results provide clear evidence that red blood cells respond to CpG DNA, both as a DAMP and a PAMP.

### 7.2. ATP

Parker and Snow [77] first showed that canine red blood cells expressed the P2X7 receptor, and its activation by ATP led to an increased cation flux. This effect was not seen when erythrocytes were incubated with other adenine nucleotides. It is notable that the P2X7 receptor is also expressed on the membranes of human erythrocytes [78], and its activation by ATP increases cation flux. More importantly, the activation of the P2X7 receptor of human red blood cells leads to phosphatidylserine exposure [79]. However, this effect was much more prominent in canine erythrocytes, perhaps due to the higher levels of P2X7 receptors [79] expressed therein. The ATP-induced phosphatidylserine exposure of canine erythrocytes was also confirmed by Faulks et al. [80], excluding other nucleotides as triggers for this effect. Another important finding came from the study of Sophocleous et al. [81], who reported that the P2X7 activation-induced phosphatidylserine exposure of canine red blood cells was not altered during cellular aging. This may indicate that P2X7 is not expelled from the membrane through vesiculation.

### 7.3. Extracellular Histones

In 2014, Semerano et al. [82] reported that extracellular histones induced phosphatidylserine exposure on human erythrocytes. This effect was mainly mediated by histone 4 (H4) and led to the increased activation of thrombin. Later, another research group found that extracellular histones triggered anemia and increased erythrocyte fragility and tendency to aggregate [83]. The results of Semerano et al. were confirmed by Yeung et al. [84], who reported histone-induced phosphatidylserine exposure on red blood cells. They found that extracellular histones induced calcium influx, generation of reactive oxygen species, and activation of caspase-3. Remarkably, these effects were attenuated with the pre-treatment of red blood cells with a neutralizing antibody for TLR2. Other investigators also found that all histones could bind to erythrocytes, albeit to different extents [85]. All the extracellular histones induced hemoglobin release and generation of microvesicles with externalized phosphatidylserine. These results could be mediated by the opening or formation of ion pores by histones [85].

### 7.4. Lipopolysaccharides (LPS)

Lipopolysaccharides can induce dramatic changes in the conformation of the erythrocyte membrane proteins. This effect is mainly attributed to the hydrophobic interactions of LPS with lipids and proteins, which can subsequently influence the protein–lipid interaction, thus disrupting the red blood cell cytoskeleton and membrane stability [86]. These results were recapitulated in later studies, where LPS was found to increase erythrocyte osmotic fragility and decrease lipid fluidity [87]. More recently, Brauckmann et al. [88] reported that LPS could provoke hemolysis with direct interaction with washed red blood cells. Hence, these studies support the idea of LPS-induced physicochemical changes on erythrocytes, which could result in hemolysis.

### 7.5. Amyloids

First, Nicolay et al. [89] showed that the incubation of human red blood cells with amyloid beta (1–42) induced sphingomyelin hydrolysis and phosphatidylserine exposure. This effect was amplified by the depletion of intracellular Cl^−^. Other researchers reported that amyloid Aβ (25–35) downregulated the activity of several glycolytic enzymes and upregulated the Na^+^/K^+^ ATPase activity of rat erythrocytes in a cellular age-dependent manner [90]. The latter results possibly hold for human red blood cells too. Aβ amyloid can uncouple the erythrocyte metabolism from oxygen saturation [91], perhaps through the trimeric G protein activation-induced activation of adenylic cyclase and caspase 3 [92]. This pathway also results in the inhibition of ATP release from red blood cells [92]. Further studies identified protein kinase C and nitric oxide as important constituents of this pathway [93,94]. Apart from the regulation of metabolism, this pathway also influences erythrocyte morphology [95].

## 8. Red Blood Cells Could Connect Non-Alcoholic Fatty Liver Disease with the Risk of Atherothrombotic Strokes

NAFLD severity is associated with an increased risk of stroke [95]. Interestingly, the red blood cells of NAFLD patients present characteristics that are involved in the mechanisms of atherothrombosis. Our group has recently shown that erythrocytes obtained from NAFLD patients exhibited increased membrane cholesterol, sphingosine, bound chemokine MCP1 [96], reduced levels of sphingomyelin [97], and CD47 [96], while also increasing the release of MCP1 [98] and the sustained release of sphingosine-1 phosphate and lysophosphatidic acid [98]. Previously, other researchers showed that erythrocytes of NAFLD patients and animal models also exhibited increased phosphatidylserine exposure and reactive oxygen species [99]. Thus, we speculate that erythrocytes of NAFLD patients could partially expose patients to a greater risk for the development of atherothrombotic stroke.

## 9. Future Perspectives

Currently, erythrocytes have been investigated in the context of hemorrhagic stroke. Ni et al. first showed that cerebral hematoma was accompanied by erythrophagocytosis. Subsequently, the injection of blood lacking CD47 resulted in reduced swelling and neurological decline [74]. Interestingly, blocking CD47 resulted in higher erythrophagocytosis by microglial cells accompanied by an ameliorated clinical outcome in an experimental animal model of intracerebral hemorrhage [100]. Therapeutically, bexarotene, a retinoid acid x receptor agonist, favors erythrophagocytosis and hence, hematoma clearance [101].

Another study also implicated the role of exposed erythrocyte phosphatidylserine in brain hematoma clearance. Importantly, the loss of receptors that recognize exposed phosphatidylserine and initiate erythrophagocytosis was associated with poor outcomes in an experimental model. Clinically, higher levels of circulating AXL receptors (which recognize exposed phosphatidylserine) are associated with negative prognoses [101].

Regarding atherothrombotic strokes, the presence of erythrocytes in thrombi, while associated with density, is also associated with better handling during mechanical thrombectomy [63]. We speculate that a better understanding of the contribution of red blood cells in atherothrombotic strokes could unveil novel therapeutic targets.

## 10. Conclusions

Several risk factors for the development and progression of atherothrombotic strokes can act through the disruption of the erythrocyte normal function. Glucotoxicity, lipotoxicity, and hyperhomocysteinemia induce erythrocyte death and oxidative stress. This can culminate in endothelial dysfunction, thrombus formation and stabilization, and hypoxia. Erythrocyte arginase, oxidative stress, and biphosphoglycerate mutase could represent therapeutic targets for atherothrombotic strokes.

## Figures and Tables

**Table 1:** Summary of the mechanisms underlying the response of red blood cells to pathogen- and damage-associated molecular patterns.

Damp/Pamp	Receptor	Effect
mtDNA	TLR9	Saturation of the scavenging capacity of red blood cells Morphological changes on erythrocytes Erythrophagocytosis Inflammation
CpG DNA	TLR9	Saturation of the scavenging capacity of red blood cells Morphological changes on erythrocytes Erythrophagocytosis Inflammation
ATP	P2X7	Increased cation flux Phosphatidylserine exposure Phosphatidylserine exposure and release of microparticles (rat erythroleukemia cells)
Extracellular histones	TLR2?	Phosphatidylserine exposure Increased erythrocyte aggression and fragility Calcium influx Generation of reactive oxygen species Activation of caspase-3 Hemoglobin release Generation of microvesicles with externalized phosphatidylserine
LPS	-	Important changes in the conformation of the erythrocyte membrane proteins Increased erythrocyte osmotic fragility Decreased lipid fluidity Hemolysis
Amyloids	-	Sphingomyelin hydrolysis Phosphatidylserine exposure Downregulates the activity of several glycolytic enzymes Upregulates the Na^+^/K^+^ ATPase activity Activation of adenylic cyclase Activation of caspase-3 Inhibition of ATP release Influences erythrocyte morphology

ATP: adenosine triphosphate; CpG DNA: cytosine-guanine deoxyribonucleic acid; LPS: lipopolysaccharide; mtDNA: mitochondrial deoxyribonucleic acid; P2X7: purinergic receptor 2x7; TLR2: toll-like receptor 2; TLR9: toll-like receptor 9.

## Data Availability

All data discussed within this manuscript are available on PubMed.

## Abbreviations

2,3-BPG	2,3 biphosphoglycerate
ADP	adenosine diphosphate
AMPK	adenosine monophosphate-dependent kinase
ATP	adenosine triphosphate
ADMA	asymmetric dimethylarginine
BPG mutase	Biphosphoglycerate mutase
CD47	cluster of differentiation 47
CER	ceramide
DALYs	disability-adjusted life-years
DAMP	damage-associated molecular patterns
ERK1/2	Extracellular signal-regulated kinase 1/2
FASR	FAS cell surface death receptor
FASL	FAS ligand
I-CAM	intercellular adhesion molecule 1
IL-8	interleukin 8
IL-17	interleukin 17
IFN-γ	interferon-γ
KA	kynurenic acid
LPS	lipopolysaccharide
MGF-E8	milk fat globule-EGF factor 8 protein
MCP1	monocyte chemoattractant protein 1
mtDNA	mitochondrial DNA
NET	neutrophil extracellular traps
NADPH	nicotinamide adenine dinucleotide phosphate
NF-Κβ	nuclear factor kappa-light-chain-enhancer of activated B cells
NO	nitric oxide
PAMP	pathogen-associated molecular patterns
PKCζ	protein kinase C ζ
ROS	reactive oxygen species
S1P	sphingosine 1-phosphate
SK1	Sphingosine kinase 1
Th17	T helper cells 17
TLR2	toll-like receptor 2
TLR9	Toll-like receptor 9
V-CAM	vascular cell adhesion protein
VSMC	vascular smooth muscle cell
